# LASSO-Based Pattern Recognition for Replenished Items With Graded Responses in Multidimensional Computerized Adaptive Testing

**DOI:** 10.3389/fpsyg.2022.881853

**Published:** 2022-06-17

**Authors:** Jianan Sun, Ziwen Ye, Lu Ren, Jingwen Li

**Affiliations:** ^1^College of Science, Beijing Forestry University, Beijing, China; ^2^Department of Statistics, Columbia University, New York, NY, United States; ^3^School of Mathematical Sciences, Beihang University, Beijing, China

**Keywords:** multidimensional graded response model, multidimensional computerized adaptive testing, replenished items, item-trait pattern recognition, LASSO, weighted BIC

## Abstract

As a branch of statistical latent variable modeling, multidimensional item response theory (MIRT) plays an important role in psychometrics. Multidimensional graded response model (MGRM) is a key model for the development of multidimensional computerized adaptive testing (MCAT) with graded-response data and multiple traits. This paper explores how to automatically identify the item-trait patterns of replenished items based on the MGRM in MCAT. The problem is solved by developing an exploratory pattern recognition method for graded-response items based on the least absolute shrinkage and selection operator (LASSO), which is named LPRM-GR and facilitates the subsequent parameter estimation of replenished items and helps maintaining the effectiveness of item replenishment in MCAT. In conjunction with the proposed approach, the regular BIC and weighted BIC are applied, respectively, to select the optimal item-trait patterns. Simulation for evaluating the LPRM-GR in pattern recognition accuracy of replenished items and the corresponding item estimation accuracy is conducted under multiple conditions across different numbers with respect to dimensionality, response-category numbers, latent trait correlation, stopping rules, and item selection criteria. Results show that the proposed method with the two types of BIC both have good performance in pattern recognition for item replenishment in the two- to four-dimensional MCAT with the MGRM, for which the weighted BIC is generally superior to the regular BIC. The proposed method has relatively high accuracy and efficiency in identifying the patterns of graded-response items, and has the advantages of easy implementation and practical feasibility.

## Introduction

With the rapid development of modern assessment theory and computer technology, computerized adaptive testing (CAT; e.g., Wainer, 2000) has become a hot issue during the past several decades. The purpose of CAT is to understand the potential characteristics of examinees as accurately as possible. Constructing the optimal test for each examinee requires modern assessment theory such as item response theory (IRT) and well-established latent variable models (e.g., Embretson and Reise, 2000; Reckase, 2009; Bartholomew et al., 2011). In psychometrics, a wide variety of psychological scales are designed to measure multiple latent traits within the framework of multidimensional item response theory (MIRT). Since the IRT-based CAT has received a lot of attention from psychometricians, the proposed method in this paper is developed within the framework of the MIRT-based CAT, which is briefly referred to as multidimensional CAT (MCAT) for simplicity. A non-negligible aspect of multidimensional items fitted by MIRT models is the corresponding relationship between the items and multiple traits measured by the overall scale, which can be simply named item-trait patterns (Sun and Ye, 2019). Specifically, item-trait pattern represents the appropriate set of the latent traits that are closely associated with an item. For instance, if a scale measures *K* traits, the true item-trait pattern of the *j*th item can be formulated as a vector: **Q**_*j*_ = (*q*_*j1*_,…, *q*_*jK*_)^T^. If the *k*th trait is measured by the item, *q*_*jk*_ = 1; else, *q*_*jk*_ = 0 (*k* = 1, …, *K*). Misspecification of the item-trait patterns of the new or replenished items fitted by MIRT models may lead to serious lack-of-fit and faulty assessment.

Item replenishment is an essential part of MCAT in view of test security and reliability. In fact, the MCAT developed on MIRT models has attracted growing attention in psychometrics. An important component of MCAT is the quality of the item pool of MCAT, the statistical perspective of which is greatly affected by the calibration of item parameters. As pointed out by Wainer and and Mislevy (1990), CAT is usually administered to examinees at frequent time intervals, some operational items in the item pool may become obsolete or overexposed over time, and the frequently used items should be replaced by replenished items considering the following reasons such as the item exposure control, safety, fairness, and reliability of the test. Also, for the MCAT, it is often encountered that some operational items in the item pool will no longer be applicable due to the similar reasons as above, so the item pool needs updating in a timely manner. Item-trait pattern and parameter estimate jointly convey the core statistical information of a multidimensional replenished item, and the identification of the item-trait pattern undoubtedly affects estimation accuracy and application of the item. For the multidimensional paper-and-pencil test, a latent variable selection method for the MIRT models *via* the least absolute shrinkage and selection operator (LASSO) is proposed by Sun et al. (2016). The research assumed the latent traits were entirely unknown and formulated the item-trait pattern recognition problem as the latent variable selection. For replenished items of MCAT, although domain experts can design which latent traits are measured by the overall MCAT, the patterns in the pool are difficult to know precisely and efficiently. Moreover, the parameter scaling consistency among all items should be ensured. Therefore, manually identifying the patterns of replenished items may face the problem of not only the heavy workload but also the risk of inconsistent scaling of item parameters. For the above-mentioned reasons, one topic of item pool replenishment for the MCAT is the timely and accurate identification of item-trait patterns for replenished items (Sun and Ye, 2019). The specific dimensions measured by an item can be briefly represented as the item-trait pattern, which reflects the goodness-of-fit for the item based on the designed MIRT model and closely relates to the estimation accuracy of latent traits. The misspecification of item-trait patterns may lead to serious lack-of-fit and faulty assessment (e.g., Reckase, 2009; Sun et al., 2016; Sun and Ye, 2019). For the MCAT based on MIRT models, it is therefore appropriate to conduct the study on identifying the patterns for replenished items.

A variety of models have been proposed in the MIRT framework. Compensatory and non-compensatory models are seen as two major types of parametric MIRT models. The most commonly used compensatory MIRT models in MCAT are multidimensional two parameter logistic model (M2PLM; McKinley and Reckase, 1982), multidimensional three parameter logistic model (Reckase and McKinley, 1991), multidimensional partial credit model (Kelderman and Rijkes, 1994), multidimensional graded response model (MGRM; Muraki and Carlson, 1995). For the compensatory MIRT model, M2PLM, Sun and Ye (2019) propose a pattern recognition method based on LASSO to detect the optimal item-trait patterns of the replenished items. The method is named LPRM, which is applicable to the MCAT with dichotomous items and has been shown to make the precise and effective detection of item-trait patterns for replenished items in the multidimensional item pool. The LPRM utilizes the online feature of the MCAT to successfully achieve the goal of automatic scaling for replenished and operational items in the item pool, so that the interpretability of replenished items in terms of goodness-of-fit can be improved. In addition, the method for dichotomous items is well viable because it is sufficiently compatible and adaptable to the conventional design of the MCAT. Consequently, the LPRM is well-suited for dichotomous items with M2PLM, which can save time cost compared to manual identification.

The research scenario of LPRM can be expanded, such as establishing a pattern recognition framework for the MCAT with polytomous items. Note that for the multidimensional graded-response data, the MGRM is an essential compensatory MIRT model for the paper-and-pencil test or the MCAT (e.g., Jiang et al., 2016; Depaoli et al., 2018; Tu et al., 2018; Wang et al., 2018a,Wang et al., 2019; Nouri et al., 2021), so it is necessary to extend the item-trait pattern recognition idea for the items with dichotomous responses to those with graded responses. Moreover, the LPRM was developed directly based on the M2PLM. With the aim of solving the pattern recognition problem for the MCAT based on the MGRM, although the general idea of the LPRM can be similarly used, it is still necessary to detail how to extend the specific approach for the model such as designing the MCAT scenarios, constructing the corresponding L_1_-regularized optimization and deriving the algorithm, and exploring how to choose the optimal patterns for replenished items. Based on these considerations, this paper highlights the pattern recognition for the replenished items with graded responses and proposes the LPRM-GR for it.

The rest of this paper is organized as follows. Section “Methodology” gives a brief introduction to the research background in several aspects: two compensatory MIRT models, the LASSO and the pattern recognition idea for dichotomous items as well as the LPRM; then the section introduces the LPRM-GR in detail for illustrating how to detect the optimal item-trait patterns of replenished items based on the MGRM in MCAT. Section “Simulation” evaluates the performance of the proposed method in pattern recognition by simulation. Section “Conclusion and discussion” summarizes the conclusion and makes the further discussion of the study.

## Methodology

### Two Compensatory Multidimensional Item Response Theory Models

#### Multidimensional Two-Parameter Logistic Model

One widely used compensatory MIRT model for dichotomous items is the M2PLM. Sun and Ye (2019) investigate the efficacy of the M2PLM based on the LASSO for detecting item-trait patterns of the replenished items in MCAT, and showed that the LASSO significantly contributes to the detection of optimal patterns of replenished items in MCAT. For a dichotomous-response test, the M2PLM is here briefly introduced: assume that the abilities of examinees follow a normal distribution. The probability of an examinee with abilities θ correctly answering the *j*th item defined by the M2PLM (e.g., Reckase, 2009) is


(1) $$P\left(U_{j}=1|\theta \right)=\frac{1}{1+\exp\left[-\left({\text{a}}_{j}^{T}\,\theta +b_{j}\right)\right]},$$

where *U*_*j*_ is a binary random variable for the response of the examinee to the *j*th item. The discrimination parameters of the *j*th item are denoted as the vector: **a**_*j*_. The intercept parameter of the *j*th item is denoted as *b*_*j*_.

#### Multidimensional Graded Response Model

For a polytomous-response test, MGRM is one of the most popular MIRT models discussed by psychometricians in the past decades. As a polytomous extension of M2PLM, MGRM can appropriately analyze and score ordered categorical data, such as in a Likert-type rating scale (e.g., Jiang et al., 2016). Assume that *N* examinees are taking a multidimensional test with *J* graded-response items measuring *K* latent traits of interest. According to the logistic-form of the MGRM, the probability of the *i*th examinee to the *j*th item receiving a score of *m* or above is defined as


(2) $$P_{i\,j\,m}^{*}\left(\theta_{i}\right)=P^{*}\left(Y_{i\,j}\geq m|\theta_{i}\right)=\frac{1}{1+\exp\left[-\sum_{k=1}^{K}a_{j\,k}\,\left(\theta_{i\,k}-d_{j\,m}\right)\right]}\left(m=0,1,\ldots ,M.\right)$$

where *Y*_*ij*_ refers to the score taking an integer from 0 to *M*; {*a*_*jk*_} (*k* = 1, …, *K*) and {*d*_*jm*_} (*m* = 0, …, *M*) are discrimination parameters and boundary parameters, respectively. By denoting $b_{j\,m}=\left(-\sum_{k=1}^{K}a_{j\,k}\right)\,d_{j\,m}$ (i.e., the intercept parameter for *j* and *m*), **a**_*j*_ = (*a*_*j*1_,…,*a*_*j*K_)T and θ_*i*_ = (θ_*i*1_,…,θ_*iK*_)T (*i* = 1, …, *N*), Equation (2) is rewritten as $P_{i\,j\,m}^{*}\,\left(\theta_{i}\right)=\frac{1}{1+\exp\left[-\left(a_{j}^{\text{T}}\,\theta_{i}+b_{j\,m}\right)\right]}$. Note that the constraints should be satisfied: $P_{i\,j\,0}^{*}\,\left(\theta_{i}\right)=1$, $P_{i\,j\,\left(M+1\right)}^{*}\,\left(\theta_{i}\right)=0$, and + ∞ = *b*_*j*0_ > … > *b*_*jM*_ = −∞. With the definition of boundary function, the probability of the *i*th examinee receiving the score of *m* to the *j*th item is expressed as


(3) $$P_{i\,j\,m}\left(\theta_{i}\right)=P\left(Y_{i\,j}=m|\theta_{i}\right)=P_{i\,j\,m}^{*}\left(\theta_{i}\right)-P_{i\,j\,\left(m+1\right)}^{*}\left(\theta_{i}\right).$$

Note that MGRM is a compensatory item factor model, so here gives a brief discussion about the identifiability of the discrimination parameters of the MGRM. If the ability parameters are unknown, the MMLE/EM algorithm (e.g., Cai, 2013) is commonly used to estimate item parameters under the condition of some or all patterns are well-known. For example, Jiang et al. (2016) and Wang et al. (2018a) both assumed that the simple structures of the overall items are satisfied for estimating the item parameters for graded-response items *via* the EM algorithm: for each item, only one element of the **a** vector was non-zero, and every dimension was represented by an equal number of items. Following the usual confirmatory factor analysis, other reasonable structures for the overall items based on the MGRM can also be specified before estimation. However, if both the ability parameters and most of the item-trait patterns are unknown, constraints need to be imposed on the discrimination parameters to guarantee their identifiability, which follows the usual exploratory factor analysis. The ways of giving constraints for the discrimination parameters can be various, the basic idea for which is pre-specifying the patterns of a few items so that it is possible for the other items to obtain reasonable results in terms of estimating discrimination parameters *via* the EM algorithm. For instance, one approach is to select *K* items that can measure all the *K* latent traits, and then pre-specify their item-trait patterns. More detailed discussion can be found in Béguin and Glas (2001), and Sun et al. (2016). If the abilities of the MGRM are known, it is not necessary to impose any constraint on the discrimination parameters or pre-specify any item-trait pattern for the identification of the discrimination parameters.

### Least Absolute Shrinkage and Selection Operator-Based Pattern Recognition for Dichotomous Items

The most widely used approaches of statistical variable selection in regression analysis before the 1990s are forward selection, backward elimination, stepwise selection, and ridge regression (e.g., Hoerl and Kennard, 2000). Ridge regression is also known as the regression based on L_2_ regularization. By introducing the L_2_ regularization term to the optimization problem of coefficient estimation, ridge regression produces shrinkage of the size of the regression coefficients to mitigate the estimation problem due to multicollinearity in linear regression. Ridge regression improves prediction accuracy more efficiently than the previous variable selection approaches, especially for the linear models suffering a large number of covariates and multicollinearity problems. The LASSO optimization is one of the members of the penalized least squares, the idea of which can be extended naturally to likelihood-based models (e.g., Tibshirani, 1996; Fan and Li, 2001). The LASSO penalty corresponds to a Laplace prior, which expects many coefficients to be close to zero, and a small subset to be larger and non-zero, so the LASSO tends to pick relatively simple combination structure from the alternative predictors (Tibshirani, 1996; Friedman et al., 2007). Specifically, the LASSO is usually labeled as the L_1_-constrained fitting or the L_1_ regularization for regression models. The optimizations based on the ridge regression and the LASSO can both consider the complexity of the regression models. In contrast to ridge regression, the LASSO can furtherly improve model interpretability by minimizing the sum of squared errors with a bound on the sum of the absolute values of the regression coefficients, which tends to produce some coefficients directly to be zero (Tibshirani, 1996). To be brief, the LASSO is a regression method that involves penalizing the absolute size of the regression coefficients and is considered as one of the most popular methods for variable selection in multivariate regression analysis. Two essential features of the LASSO are the shrinkage and selection of regression variables.

For MIRT models, the problems of variable selection based on the LASSO have ever been discussed in the research of latent variable selection (Sun et al., 2016). For the pattern recognition problem for replenished items in MCAT based on the M2PLM, the LPRM based on the LASSO is proposed by Sun and Ye (2019). Penalized regression methods can also be taken into consideration for analyzing the data of large-scale surveys with missing categorical predictors, which inspires a new perspective for the effective and feasible treatment of missing data in the field of large-scale testing (e.g., Yoo, 2018; Yoo and Rho, 2020, 2021).

The LPRM generally collects the information in terms of examinees’ ability estimates and responses to replenished items *via* the MCAT to prepare for the item-trait pattern recognition, uses the LASSO to obtain alternative patterns of replenished items, and finally applies the regular Bayesian information criterion (BIC) to select the optimal patterns from the alternatives. Details of the roles played by the MCAT and the LASSO in LPRM can be understood by reviewing the three steps of the LPRM. The first step is to operate the MCAT measuring *K* latent traits of interest with *N* examinees. A certain number of operational items are selected from all the operational items by the given item selection method in MCAT. A fixed number of replenished items are specified among all the replenished items in the item pool. The appropriate number of administered replenished items can be designed to make sure that the length of the MCAT is workable for examinees. For instance, if the number of replenished items is *J*_1_, *N* examinees can be divided into 5 groups and every *J*_1_/5 replenished items are answered by each group. Note that the replenished items are also assumed to be consistent with the operational ones so that the examinees share the same test motivation for the two item types. All answers are recorded and scored, which aims to get valuable information in terms of both examinees’ ability estimates and the responses to replenished items. Secondly, the LASSO or L_1_-regularized optimization for the *j*th replenished item is formulated in Equation (4), which can be considered the essential part of the second step of the LPRM. Because θ*_*i*_* and **a***_*j*_* in Equation (2) play the role of covariates and coefficients, respectively, the sparsity of **a***_*j*_* indicates the item-trait patterns. That is, if ${\hat{a}}_{j\,k}>0$, then *q*_*jk*_ = 1; else *q*_*jk*_ = 0.


(4) $$\min_{b_{j},a_{j\,1},\ldots ,a_{j\,K}}\left\{-l\,\left(b_{j},a_{j\,1},\ldots ,a_{j\,K};{\text{Y}}_{j},\Theta \right)+\lambda \,\sum_{k=1}^{K}\left|a_{j\,k}\right|\right\}.$$

Here continuing the notations in Equation (1), the abilities in MCAT are denoted by Θ = (θ_*ik*_)*N*×*K*. In Equation (4): *l*(*b*_*j*_,*a*_*j*1_,…,*a*_*jK*_;**Y**_*j*_,Θ)is the log-likelihood based on the observed data, **Y**_*j*_, and the latent variables, Θ. Note that for the LPRM, Θ can be substituted by ability estimates. The L_1_-norm of discrimination parameters for the *j*th item is denoted as $\sum_{k=1}^{K}\left|a_{j\,k}\right|$. The tuning parameter (also named regularization parameter), λ, mainly controls the sparsity of the discrimination parameters. A group of non-negative values of λ is given to produce adequate alternative patterns according to the discrimination parameters, which are obtained by the corresponding L_1_-regularized optimizations for alternative values of λ. Coordinate descent algorithm can be used to solve the optimizations (e.g., Friedman et al., 2010). Thirdly, the regular BIC is applied to choose the optimal item-trait patterns of replenished items from the alternative ones that are obtained in the second step.

Primary findings of the LPRM that may enlighten the study of this paper are summarized as follows. Firstly, for the replenished items in the MCAT item pool based on M2PLM, the correct specification rates for which patterns detected by the LPRM are above 80% and almost above 90% in the simulation of Sun and Ye (2019). Because the true abilities are unknown in practice, the LPRM can generally get comparatively sufficient ability information from ability estimates and select good patterns for replenished items. Secondly, the item parameters of the replenished items are estimated based on the patterns identified by the LPRM with the aim of investigating how the recognition accuracy of the patterns influences the estimation accuracy of item parameters, the finding for which is that the former affect the latter positively. Thirdly, the operational items with high discrimination can help the LPRM yield comparatively good performance in identifying patterns and getting the estimates of discrimination parameters. Fourthly, in practice, there is a trade-off for the LPRM to choose which of the variable-length MCAT and the fixed-length MCAT should be used, and the possible factors include the sizes of discrimination parameters, computing efficiency of the two stopping rules and other practical considerations about test safety and item exposure.

### Least Absolute Shrinkage and Selection Operator-Based Pattern Recognition for Graded-Response Items

In this section, we propose the framework of the LASSO-based pattern recognition method for the item pool with graded response items in the MCAT. The proposed method is named LPRM-GR, the aim of which is to effectively give an appropriate polytomous extension of the LPRM: specially developed for the MCAT constructed based on the MGRM. The detailed steps of the proposed method in this paper are illustrated as follows.

Step 1. Consider a situation that *K* latent traits of interest are measured by an MCAT item pool, which includes fixed numbers of operational and replenished items. When an MCAT with *N* examinees is organized, appropriate numbers of operational and replenished items can be designed in the test for balancing the goal of individual measurement and collection of responses to replenished items. For instance, the numbers of two types of items in the item pool are *J*_0_ and *J*_1_, respectively. At the same time, assume that it has been ensured that the examinees have the same motivation for both types of items in the test. Note that the test length here should also take into account a combination of considering different types of stopping rules and avoiding fatigue effects. Note that the design for the LPRM-GR here is similar to the first step of the LPRM.

Step 2. For all the replenished items in the item pool of the MCAT, a range of non-negative values of the tuning parameter for the LASSO, λ_1_,…,λ_*W*_, should be given for providing various shrinkage effects for discrimination parameters of the MGRM. Specifically, the L_1_-regularized optimization constructed for the *j*th item with a given λ_*w*_ is defined as


(5) $$\min_{b_{j\,1},\ldots ,b_{j\,\left(M-1\right)},a_{j\,1},\ldots ,a_{j\,K}}\{-l\left(b_{j\,1},\ldots ,b_{j\,\left(M-1\right)},a_{j\,1},\ldots ,a_{j\,K},{\text{Y}}_{j},\hat{\Theta }\right)+\lambda_{w}\sum_{k=1}^{K}|a_{j\,k}|\},$$

where the true abilities are denoted as Θ = (θ_*ik*_)N×K, and their estimates accordingly as $\hat{\Theta }$. **Y**_*j*_ = (*Y*_1*j*_,…,*Y*_*Nj*_)*T* is the vector of responses to the *j*th item for all examinees, and therefore $-l\,\left(b_{j\,1},\ldots ,b_{j\,\left(M-1\right)},a_{j\,1},\ldots ,a_{j\,K},{\text{Y}}_{j},\hat{\Theta }\right)$ represents the log-likelihood of **Y**_*j*_ and $\hat{\Theta }$ for the MGRM. The L_1_-norm penalty of **a**_*j*_ for the *j*th item is expressed as $\sum_{\text{k}=1}^{K}\left|a_{j\,k}\right|$. Generally, larger tuning parameter tends to cause more sparse discrimination parameters, so λ_*w*_ directly influences the shrinkage effect of ${\hat{\text{a}}}_{j}$. The convexity of the L_1_-norm penalized negative log-likelihood function in Equation (5) can be easily proven by convex function properties (e.g., Boyd and Vandenberghe, 2004; Nocedal and Wright, 2006; Hastie et al., 2015), so the coordinate descent algorithm (Friedman et al., 2007, 2010) can be used to solve the optimization. Specifically, set the relatively small value of λ for which the entire vector **a**_*j*_ is equal to zeros. The estimated abilities,$\hat{\Theta }$, and observed response data, **Y**_*j*_, are inputted; the solution with respect to item parameters, (*b*_*j*1_,…,*b*_*j*(*M*−1)_,*a*_*j*1_,…*a*_*jK*_), to the LASSO optimization for the MGRM based on the setting of λ is updated coordinate by coordinate, the details for which are illustrated in the Appendix section. Thus, the solutions to the optimizations with a decreasing sequence of λ can be obtained respectively. That is, for *W* values of the tuning parameter, the discrimination parameter estimates,${\hat{\text{a}}}_{\text{j}}^{\left(1\right)}$, …, ${\hat{\text{a}}}_{\text{j}}^{\left(W\right)}$, can be obtained *via* the algorithm and the alternative item-trait patterns of the *j*th item is accordingly obtained. Denote the true item-trait pattern for *j* as **Q***_*j*_* = (*q*_*j*1_, …, *q*_*jK*_)*^T^*, and *q*_*jk*_ = 1 represents the *k*th ability is measured by that item, while *q*_*jk*_ = 0 represents the opposite case. Denote ${\hat{\text{Q}}}_{j}^{\left(w\right)}=\left({\hat{q}}_{j\,1}^{\left(w\right)},\ldots ,{\hat{q}}_{j\,K}^{\left(w\right)}\right)$ as the estimated item-trait pattern of Equation (5). If ${\hat{a}}_{j\,k}>0$, then *q*_*jk*_ = 1; else *q*_*jk*_ = 0. The optimal item-trait pattern is denoted by ${\text{Q}}_{j}^{*}=\left(q_{j\,1}^{*},\ldots ,q_{j\,K}^{*}\right)$. For this research, because necessary ability estimates can be obtained based on the operational items in MCAT, so the patterns of replenished items for the MGRM can be directly estimated (identified) *via* optimizations of the LPRM-GR without factor rotation.

Step 3. Apply an appropriate selection index to choose the optimal item-trait patterns for the replenished items from the alternative patterns. As discussed in Sun and Ye (2019), the goodness-of-fit for the M2PLM to the data can be appropriately measured by the regular BIC and it is implemented for finding the optimal patterns from the alternatives, for which the decision rule is minimizing the alternatives in terms of BIC. In this research, the regular BIC for the replenished items with a given λ can be defined as


(6) $$B\,I\,C=-2\,\sum_{j=1}^{J_{1}}\sum_{i=1}^{N}\log\left({\hat{l}}_{i\,j}\right)+n_{p}\,\log\left(N\right),$$

where *n*_*p*_ represents the number of item parameters obtained by the result of the LASSO optimization such as that in Equation (5); *N* represents the number of examinees and ${\hat{l}}_{i\,j}$ calculates the log-likelihood based on the *i*th examinee’s ability estimate. The decision rule is to minimize the regular BIC based on a combination of alternative patterns with different λ values.

Note that for the LRPM-GR, it could also be tried to adjust the regular BIC as the weighted BIC in order to furtherly improve the precision of pattern selection from the ones in the above steps. There have been a few studies highlighting the effects of response distribution in parameter estimation and pattern selection (e.g., King and Zeng, 2001; Hu and Zidek, 2002, 2011). Hu and Zidek (2002) addressed the challenges of imbalanced classes of the population problem in maximum likelihood estimation. They show that the unbalanced distribution of responses did influence the parameter estimation accuracy and pointed out that appropriate weights enable researchers to represent the degree to which the information from the populations should be used in fitting the likelihood. Furthermore, Hu and Zidek (2002) believe that the best choice of the weights will depend on the context, and the weighted likelihood with data-dependent weights can be referred to as adaptive weighted likelihood. Thus, the weighted BIC for a given λ is here constructed in manners of adaptive weighted likelihood to assist in improving the effects of the response distribution (or the observed information of the examinee populations) in the pattern selection process:


(7) $$B\,\tilde{I}\,C=-2\,\sum_{j=1}^{J_{1}}\sum_{i=1}^{N}\nu_{i\,j}\,\log\left({\hat{l}}_{i\,j}\right)+n_{p}\,\log\left(N\right),$$

where the weights {ν_*ij*_} are based on the score of the examinee as well as the corresponding frequency with which examinees scored at the same response level: ν_*ij*_ = η_*j*,*Y*_*ij*__. That is, if *Y*_*ij*_ = *m*(*m* = 0, …, *M*), then ν_*ij*_ = η_*jm*_, where $\eta_{j\,m}=\sum_{i=1}^{N}\frac{I\left(Y_{i\,j}=m\right)}{N}$.

So the weighted BIC modifies the log-likelihood by weighting the likelihood based on the above frequencies. The characteristic of the specific form of the weighted BIC here is that the weights are easier to obtain and less computationally intensive. Similar to Hu and Zidek (2002)’s point for the weighted BIC, the practical meaning of the weights in the weighted BIC here can be understood as enabling the contribution of observed responses to the likelihood of the criterion to be enhanced; that is, the likelihood could be emphasized by means of giving the degree of the information from the examinee populations. Based on the above considerations, the regular BIC and weighted BIC were, respectively, applied in the simulation section to choose the optimal item-trait patterns from the alternatives for all the replenished items.

The shrinkage effect of the LASSO leads to controllable bias to the item parameter estimation. The estimated item parameters obtained from the L_1_-regularized optimizations should not be directly used to calculate the likelihood in the regular BIC or weighted BIC. Thus, the calibration of item parameters for all the replenished items should be re-estimated based on the alternative patterns from the optimizations with different λ values, which is similar to the treatment for the simplified relaxed LASSO (Meinshausen, 2007; Hastie et al., 2017). The response data can be refitted by the MGRM based on each of the alternative patterns *via* the algorithm of path-wise coordinate descent, the corresponding optimization for which is simply the estimation based on likelihood without involving the L_1_-norm penalty. The decision rule is to minimize the regular BIC or weighted BIC based on alternative patterns with different λ values.

## Simulation

Studies 1, 2, 3 corresponding to the two-, three-, and four-dimensional MCAT item pools were conducted to evaluate the performance of the LPRM-GR for detecting the optimal item-trait patterns. In each study, there were 16 conditions considered, which were across different latent trait correlation (i.e., Σ), response categories for the graded-response data (i.e., *M*+1), test lengths, and item selection criteria. Specifically, independent and correlated latent traits were designed, respectively, for generating response data based on the MGRM. Two types of response categories were considered for the MGRM: *M*+1 = 3 and 4. The fixed-length and variable-length stopping rules were designed, respectively, for the MCAT. Two types of item selection criteria were considered for the MCAT procedure of the LPRM-GR: Bayesian D-optimality and Bayesian A-optimality (e.g., Berger and Veerkamp, 1994; van der Linden, 1999; Mulder and van der Linden, 2009). Besides, the regular BIC and weighted BIC were applied by the LPRM-GR, respectively, to choose the optimal item-trait patterns from the alternatives for replenished items. For each condition in the simulation study, 50 replications were conducted. The computational codes were written in the R software.

### Item Pool and Data Design

#### Item Pool Generation

For each of the three studies, discrimination parameters ({*a*_*jk*_}) were randomly sampled from the uniform distribution, *U*(0.7,1.5); boundary parameters ({*d*_*jm*_}) were drawn from *U*[−2,2] and each was uniformly distributed along with an equidistant interval within this range for each item (e.g., Jiang et al., 2016). Specifically, boundary parameters for *M*+1 = 3 were randomly sampled from *U*[−2,−0.67], *U*[−0.67,0.67], and *U*[0.67,2], respectively. Boundary parameters for *M*+1 = 4 were randomly sampled from *U*[−2,−1], *U*[−1,0], *U*[0,1], and *U*[1,2], respectively.

In Study 1, each two-dimensional item pool had *J*_0_ = 900 operational items, of which 300 items measured the first trait, another 300 items measured the second trait, and the remaining 300 items measured both the two traits, so 3 types of patterns were designed for the study. Each item pool of Study 1 had *J*_1_ = 30 replenished items, of which 10 items measured the first trait, another 10 items measured the second trait, and the rest measured both the two traits. Similarly, since three latent traits were measured by each item pool of Study 2, there were seven types of patterns designed. Each item pool of the study consisted of *J*_0_ = 910 operational items and *J*_1_ = 35 replenished items, of which every 130 operational items and every five replenished items corresponded to one of the seven types of patterns. In Study 3, each four-dimensional item pool had *J*_0_ = 900 operational items and *J*_1_ = 30 replenished items, of which every 60 operational items and every two replenished items had one of the 15 types of patterns.

#### Response Data Generation

For all of the three studies, the true abilities of *N* = 4,000 examinees are randomly sampled from two multivariate normal distributions with N(0,Σ_*i*_), (*i* = 1,2), respectively. For the two-dimensional study, covariance matrices were Σ_1_ = $\left(\begin{array}{cc} 1 & 0\\ 0 & 1 \end{array}\right)$ and Σ_2_ = $\left(\begin{array}{cc} 1 & 0.3\\ 0.3 & 1 \end{array}\right)$; for the three-dimensional study, those were Σ_1_ = $\left(\begin{array}{ccc} 1 & 0 & 0\\ 0 & 1 & 0\\ 0 & 0 & 1 \end{array}\right)$ and Σ_2_ = $\left(\begin{array}{ccc} 1 & 0.2 & 0.3\\ 0.2 & 1 & 0.5\\ 0.3 & 0.5 & 1 \end{array}\right)$; for the four-dimensional study, those were Σ_1_ = $\left(\begin{array}{cccc} 1 & 0 & 0 & 0\\ 0 & 1 & 0 & 0\\ 0 & 0 & 1 & 0\\ 0 & 0 & 0 & 1 \end{array}\right)$ and Σ_2_ = $\left(\begin{array}{cccc} 1 & 0.2 & 0.4 & 0.5\\ 0.2 & 1 & 0.3 & 0.5\\ 0.4 & 0.3 & 1 & 0.7\\ 0.5 & 0.5 & 0.7 & 1 \end{array}\right)$. The responses of the examinees were generated from the MGRM based on the true values of the parameters given above.

### Multidimensional Computerized Adaptive Testing Procedure in LPRM-GR

The MCAT procedure with the MGRM required by the LPRM-GR was simulated by the R package: mirtCAT (Chalmers, 2016). The estimation of examinees’ abilities based on the MGRM was obtained by the maximum a posterior (MAP), which is generally proven by numerous previous research to provide more accurate results than the method of expected a posterior. As introduced by Steps 1 and 2 of the LPRM-GR, the ability estimates from Step 1 were directly exploited for the subsequent L_1_-regularized optimization in Step 2.

#### Item Selection Criterion

Item selection is one of the key components of CAT, and frequently used item selection approaches are constructed based on Fisher Information or Kullback-Leibler Information (e.g., Wang and Chang, 2011; Wang et al., 2011; Wang et al., 2013; Tu et al., 2018). For MCAT, the item selection strategy based on Fisher information is frequently used. In this paper, the operational items were selected from the item pool by Bayesian D-optimality and Bayesian A-optimality, respectively. Bayesian A-optimality selects the *j*th item from the remaining items in the item pool by minimizing the traces of the inverse to the sum of the Fisher information matrices and a prior covariance matrix, which is formulated as


(8) $$\arg\min\left\{\text{trace}\,{\left[I_{S_{j-1}}\,\left({\hat{\theta }}^{j-1}\right)+I_{i_{j}}\,\left({\hat{\theta }}^{j-1}\right)+\Phi^{-1}\right]}^{-1}\right\},$$

where $I_{S_{j-1}}\,\left({\hat{\theta }}^{j-1}\right)$ and $I_{i_{j}}\,\left({\hat{\theta }}^{j-1}\right)$ represent the information matrices based on ${\hat{\theta }}^{j-1}$ for the *j*-1 administered items and candidate item *i*_*j*_, respectively. The prior covariance matrix for abilities is denoted as Φ. Bayesian D-optimality selects the *j*th item from the remaining items in the item pool by maximizing the determinants of the sum of the Fisher information matrices and a prior covariance matrix for abilities:


(9) $$\arg\max\left\{\det\left[I_{S_{j-1}}\,\left({\hat{\theta }}^{j-1}\right)+I_{i_{j}}\,\left({\hat{\theta }}^{j-1}\right)+\Phi^{-1}\right]\right\}.$$

#### Stopping Rules

The goal of a fixed-length stopping rule is to terminate the CAT with a predetermined length. However, it is shown that the fixed-length rule may produce less accurate results for the examinees with quite different abilities from the average difficulty level of the item pool (e.g., Wang et al., 2013). Compared with the fixed-length rule, the variable-length rule (e.g., Boyd et al., 2010) such as the standard error (SE) rule (e.g., Weiss and Kingsbury, 1984; Yao, 2013) can provide approximately equal precision for all examinees regardless of their different abilities. The SE rule simply controls that the standard error of the precision of each examinee’s ability estimate does not exceed a predetermined threshold. The SE rule is simple to implement as well as previous studies have also shown its good performance (e.g., Yao, 2013). Thus, the above two types of stopping rules were used in this study, respectively. The first was the fixed-length rule: the MCAT in the simulation was stopped at *Z*_0_ = 50 operational items for each examinee. The second was the SE rule with a maximum 100-length constraint, which aims to jointly consider approximate similar measurement precision as well as appropriate test length for the MCAT because using the SE rule alone could produce a too long test for a few examinees with quite low or high abilities (e.g., Wang et al., 2018b); specifically, the MCAT is terminated once a pre-specified estimated standard error, 0.3, along with 100-length was reached.

#### Assignment of Replenished Items

All the replenished items were divided into five groups and assigned furtherly to each group. *Z*_1_ = 6 replenished items were assigned to each group for the two-dimensional study while *Z*_1_ = 7 for the three-dimensional study. Note that assigned replenished items contained all possible patterns. For the four-dimensional study, each group was assigned *Z*_1_ = 6 replenished items, which corresponded to six different patterns, respectively. The examinees were equally divided into five groups either and answered assigned replenished items. Thus, *n* = 800 examinees were assigned to answer different replenished items.

#### Specification of Tuning Parameter

As is known, the sparsity of patterns for the LASSO can be affected by λ, so its alternative values should be given in a relatively large range to ensure the adequacy of the alternative item-trait patterns; whereas too many alternative values may produce similar patterns instead of diverse ones and cause unnecessary computing load. Therefore, dozens of equidistant cut-off points of λ are set between 0 and 120 for Step 2 of the proposed method after careful trials in simulation.

### Evaluation Criteria

#### Correct Specification Rate

To evaluate the precision effects of the LPRM-GR on pattern recognition, one intuitive index is the correct specification rate of replenished items:


(10) $$\mathrm{CSR}=\frac{1}{J_{1}\times K}\sum_{j=1}^{J_{1}}\sum_{k=1}^{K}I\left(q_{j\,k}^{*}=q_{j\,k}\right),$$

where **Q**_*j*_ = (*q*_*j*1_,…,*q*_*jK*_)*T* is the true pattern of the *j*th replenished item, and ${\hat{\text{Q}}}_{j}={\left(q_{j\,1}^{*},\ldots ,q_{j\,K}^{*}\right)}^{T}$ is the identified pattern by the LPRM-GR. As above, each element of **Q**_*j*_ takes 1 or 0, indicating the corresponding trait is or is not measured by the *j*th item. The identity function is denoted by *I*. Note that CSR falls in [0, 1], and the larger value of CSR means the higher recognition accuracy.

#### Ability Estimation Accuracy

The absolute mean error (AME) and root mean square error (RMSE) are calculated as follows to evaluate the ability estimation accuracy of the examinees in the MCAT procedure:


(11) $$\mathrm{AME}\,\left(\theta \right)=\frac{\sum_{i=1}^{N}\sum_{k=1}^{K}\left|{\hat{\theta }}_{i\,k}-\theta_{i\,k}\right|}{N\times K},$$

(12) $$\text{RMSE}\,\left(\theta \right)=\sqrt{\frac{\sum_{i=1}^{N}\sum_{k=1}^{K}{\left({\hat{\theta }}_{i\,k}-\theta_{i\,k}\right)}^{2}}{N\times K}},$$

where ${\hat{\theta }}_{i\,k}$ is the estimated ability on the *k*th dimension of the *i*th examinee, and θ_*ik*_ is the true ability for that. Smaller values of the two indices correspond to the higher estimation accuracy of abilities.

#### Item Parameter Estimation Accuracy

Similar to the evaluation indices in ability vectors, the AME is applied to measure the estimation of item parameters of replenished item parameters, which is calculated based on the patterns identified by the LPRM-GR with the estimated abilities and the true abilities, individually. The two indices of item parameters of replenished items with MGRM are computed as


(13) $$\text{AME}\,\left(a\right)=\frac{\sum_{j=1}^{J_{1}}\sum_{k=1}^{K}\left|{\hat{a}}_{j\,k}-a_{j\,k}\right|}{J_{1}\times K},$$

(14) $$\text{AME}\,\left(b\right)=\frac{\sum_{j=1}^{J_{1}}\sum_{m=1}^{M-1}\left|{\hat{b}}_{j\,m}-b_{j\,m}\right|}{J_{1}\times \left(M-1\right)}.$$

Smaller values of the two indices correspond to the higher estimation accuracy of item parameters.

### Result of Study 1

#### Pattern Recognition Accuracy of Replenished Items in Study 1

Table 1 lists the CSR values of the optimal patterns of replenished items under multiple conditions based on two types of BIC in the two-dimensional item pool. Note that the table contains the CSRs for the LPRM-GR with the estimated abilities and true abilities, and those with true abilities can be used as benchmarks.

**TABLE 1:** Correct specification rate of item-trait patterns identified by the LPRM-GR in Study 1.

Condition			LPRM-GR with ability estimates				LPRM-GR with true abilities	
				
			Regular BIC		Weighted BIC		Regular BIC (%)	Weighted BIC (%)
		
*M*+1	Latent trait correlation	Test length type	Item selection criterion					
			
			Bayesian A-optimality (%)	Bayesian D-optimality (%)	Bayesian A-optimality (%)	Bayesian D-optimality (%)		
3	Independent	Fixed-length	99.63	99.53	100	100	99.67	100
		Variable-length	99.63	99.63	100	100		
	Correlated	Fixed-length	98.33	98.40	100	100	99.60	100
		Variable-length	99.13	99.07	100	100		
4	Independent	Fixed-length	99.73	99.73	100	100	99.60	100
		Variable-length	99.63	99.73	100	100		
	Correlated	Fixed-length	98.70	98.73	100	100	99.70	100
		Variable-length	99.10	99.10	100	100		

As shown in Table 1, the CSR values with estimated abilities were generally close or even equal to their benchmarks. The LPRM-GR with estimated abilities achieved quite high CSR results, which reached even 100% for the conditions with the weighted BIC. Thus, focusing on the two types of BIC, it showed that the CSRs obtained from the LPRM-GR with the weighted BIC had better performance than those with the regular BIC.

Furthermore, Table 1 showed that for the two types of response categories, the CSR values with independent-ability conditions were equal to or slightly higher than those for correlated-ability conditions. It also indicated that Bayesian A-Optimality and Bayesian D-Optimality performed closely and had quite high CSR values. Comparing the pattern recognition accuracy of the LPRM-GR between the two types of test lengths, it was found that the CSRs are close for most conditions; the CSRs for the variable-length conditions especially with correlated abilities and selected with the regular BIC were slightly better than those for the fixed-length test conditions.

#### Ability Parameter Estimation Comparison in Multidimensional Computerized Adaptive Testing of Study 1

To investigate the effects of ability estimation in pattern recognition, the parameter estimation accuracy was given in Table 2. As expected, the AME and RMSE results of ability estimation for the variable-length test showed a significant improvement in estimation accuracy over the fixed-length test. It also shows that AME and RMSE values for Bayesian A-Optimality were generally lower than those for Bayesian D-Optimality. Although the AME and RMSE values of correlated abilities were slightly lower than those for independent abilities, the CSR values presented in Table 1 indicated that the pattern recognition accuracy for the correlated abilities was slightly lower than independent ones for the regular BIC.

**TABLE 2:** Recovery of ability parameters in Study 1.

Condition			Item selection criterion			
			
			Bayesian A-optimality		Bayesian D-optimality	
		
*M*+1	Latent trait correlation	Test length type	AME (θ)	RMSE (θ)	AME (θ)	RMSE (θ)
3	Independent	Fixed-length	0.3081	0.3865	0.3215	0.4036
		Variable-length	0.2464	0.3091	0.2570	0.3226
	Correlated	Fixed-length	0.2981	0.3741	0.3094	0.3887
		Variable-length	0.2414	0.3029	0.2512	0.3152
4	Independent	Fixed-length	0.2978	0.3733	0.3066	0.3848
		Variable-length	0.2398	0.3006	0.2440	0.3061
	Correlated	Fixed-length	0.2875	0.3607	0.2952	0.3703
		Variable-length	0.2345	0.2940	0.2384	0.2991

#### Item Parameter Estimation Accuracy of Replenished Items in Study 1

To furtherly measure the impact of detected patterns on the recovery of replenished items and for brevity, AME values of estimated item parameters under different conditions with two types of BIC are presented in Tables 3, 4. The two tables showed that the AME values of estimated discrimination parameters based on the patterns selected by the weighted BIC were slightly smaller than those by the regular BIC. Also, by the comparison of Tables 1, 3, 4, it was found that the recovery of intercept parameters was not affected by the pattern recognition accuracy as sensitively as the discrimination parameters.

**TABLE 3:** Recovery of item parameters based on patterns from the LPRM-GR with ability estimates and regular BIC in Study 1.

Condition			LPRM-GR with ability estimates			
	
*M*+1	Latent trait correlation	Test length type	Item selection criterion			
			
			Bayesian A-optimality		Bayesian D-optimality	
				
			AME (*a*)	AME (*b*)	AME (*a*)	AME (*b*)
3	Independent	Fixed-length	0.0510	0.0867	0.0516	0.0874
		Variable-length	0.0487	0.0830	0.0488	0.0835
	Correlated	Fixed-length	0.0599	0.0869	0.0609	0.0877
		Variable-length	0.0525	0.0835	0.0532	0.0838
4	Independent	Fixed-length	0.0477	0.0867	0.0486	0.0871
		Variable-length	0.0460	0.0829	0.0461	0.0833
	Correlated	Fixed-length	0.0556	0.0855	0.0556	0.0855
		Variable-length	0.0501	0.0820	0.0503	0.0823

**TABLE 4:** Recovery of item parameters based on patterns from the LPRM-GR with ability estimates and weighted BIC in Study 1.

Condition			LPRM-GR with ability estimates			
	
*M*+1	Latent trait correlation	Test length type	Item selection criterion			
			
			Bayesian A-optimality		Bayesian D-optimality	
				
			AME (*a*)	AME (*b*)	AME (*a*)	AME (*b*)
3	Independent	Fixed-length	0.0502	0.0867	0.0506	0.0875
		Variable-length	0.0479	0.0830	0.0480	0.0836
	Correlated	Fixed-length	0.0550	0.0870	0.0559	0.0878
		Variable-length	0.0502	0.0835	0.0507	0.0837
4	Independent	Fixed-length	0.0472	0.0867	0.0481	0.0872
		Variable-length	0.0453	0.0829	0.0455	0.0833
	Correlated	Fixed-length	0.0520	0.0853	0.0530	0.0855
		Variable-length	0.0479	0.0820	0.0481	0.0823

### Result of Study 2

#### Pattern Recognition Accuracy of Replenished Items in Study 2

In the three-dimensional study, the CSR values for the LPRM-GR under multiple conditions are listed in Table 5. It indicated that the results in CSR were very close to their benchmarks, except for those in the correlated-ability condition for the regular BIC. Similar to the performance in pattern recognition accuracy in Study 1, the LPRM-GR in Study 2 generally produced relatively high CSRs for the overall conditions with the two types of BIC, and it especially achieved quite high CSR for the conditions with the weighted BIC.

**TABLE 5:** Correct specification rate of item-trait patterns identified by the LPRM-GR in Study 2.

Condition			LPRM-GR with ability estimates				LPRM-GR with true abilities	
				
			Regular BIC		Weighted BIC		Regular BIC (%)	Weighted BIC (%)
		
*M*+1	Latent trait correlation	Test length type	Item Selection Criterion					
			
			Bayesian A-optimality (%)	Bayesian D-optimality (%)	Bayesian A-optimality (%)	Bayesian D-optimality (%)		
3	Independent	Fixed-length	99.62	99.64	99.94	99.94	99.67	100
		Variable-length	99.41	99.50	100	100		
	Correlated	Fixed-length	94.34	94.34	99.62	99.52	99.60	100
		Variable-length	96.61	96.59	99.85	99.89		
4	Independent	Fixed-length	99.33	99.28	100	100	99.47	100
		Variable-length	99.43	99.37	100	100		
	Correlated	Fixed-length	94.17	94.11	99.28	99.49	99.50	100
		Variable-length	96.63	96.50	99.83	99.81		

In addition, Table 5 indicated that the CSRs for the conditions with two types of response categories and the independent abilities were higher than those with the correlated abilities. Results also showed that for most cases, the tests with variable-length by either Bayesian A-Optimality or Bayesian D-Optimality for the LPRM-GR had relatively higher CSR values than tests with the length of 50 items. The two types of item selection criteria showed a slight difference in CSR.

#### Ability Parameter Estimation Comparison in Multidimensional Computerized Adaptive Testing of Study 2

Table 6 listed the AME and RMSE of the estimated abilities. It showed that the ability parameter estimation accuracy for Bayesian A-Optimality was slightly better than that for Bayesian D-Optimality. Also, it was clear that the AME and RMSE for the correlated-ability conditions were lower than those for the independent-ability conditions. Table 6 showed that the abilities could be estimated more precisely for the variable-length MCAT than that for the fixed-length case.

**TABLE 6:** Recovery of ability parameters in Study 2.

Condition			Item selection criterion			
			
			Bayesian A-optimality		Bayesian D-optimality	
		
*M*+1	Latent trait correlation	Test length type	AME (θ)	RMSE (θ)	AME (θ)	RMSE (θ)
3	Independent	Fixed-length	0.4081	0.5122	0.4163	0.5223
		Variable-length	0.3334	0.4182	0.3385	0.4251
	Correlated	Fixed-length	0.3838	0.4815	0.3897	0.4888
		Variable-length	0.3204	0.4019	0.3250	0.4078
4	Independent	Fixed-length	0.3956	0.5832	0.4012	0.5034
		Variable-length	0.3195	0.4005	0.3243	0.4066
	Correlated	Fixed-length	0.3729	0.4681	0.3782	0.4748
		Variable-length	0.3088	0.3874	0.3126	0.3923

#### Item Parameter Estimation Accuracy of Replenished Items in Study 2

The AME values of the estimated item parameters based on the patterns selected by two types of BIC, individually, were listed in Tables 7, 8. It can be inferred from the two tables and Table 5 that the estimation accuracy of intercept parameters was slightly influenced by the pattern recognition accuracy of replenished items, while the recovery of discrimination parameters was more sensitive to the pattern recognition accuracy. Besides, similar to the results of Tables 3, 4, the AMEs of the estimated discrimination parameters based on the patterns selected by the LPRM-GR with weighted BIC were smaller than those with the regular BIC.

**TABLE 7:** Recovery of item parameters based on patterns from the LPRM-GR with ability estimates and regular BIC in Study 2.

Condition			LPRM-GR with ability estimates			
	
*M*+1	Latent trait correlation	Test length type	Item selection criterion			
			
			Bayesian A-optimality		Bayesian D-optimality	
				
			AME (*a*)	AME (*b*)	AME (*a*)	AME (*b*)
3	Independent	Fixed-length	0.0532	0.1286	0.0536	0.1299
		Variable-length	0.0470	0.1121	0.0470	0.1129
	Correlated	Fixed-length	0.0834	0.1208	0.0856	0.1223
		Variable-length	0.0654	0.1089	0.0654	0.1096
4	Independent	Fixed-length	0.0515	0.1245	0.0518	0.1254
		Variable-length	0.0447	0.1086	0.0450	0.1094
	Correlated	Fixed-length	0.0807	0.1163	0.0813	0.1169
		Variable-length	0.0613	0.1054	0.0623	0.1055

**TABLE 8:** Recovery of item parameters based on patterns from the LPRM-GR with ability estimates and weighted BIC in Study 2.

Condition			LPRM-GR with ability estimates			
	
*M*+1	Latent trait correlation	Test length type	Item selection criterion			
			
			Bayesian A-optimality		Bayesian D-optimality	
				
			AME (*a*)	AME (*b*)	AME (*a*)	AME (*b*)
3	Independent	Fixed-length	0.0527	0.1287	0.0531	0.1300
		Variable-length	0.0458	0.1122	0.0460	0.1129
	Correlated	Fixed-length	0.0635	0.1220	0.0652	0.1235
		Variable-length	0.0536	0.1094	0.0538	0.1001
4	Independent	Fixed-length	0.0499	0.1246	0.0500	0.1255
		Variable-length	0.0435	0.1087	0.0436	0.1095
	Correlated	Fixed-length	0.0622	0.1174	0.0614	0.1180
		Variable-length	0.0506	0.1058	0.0509	0.1059

### Result of Study 3

#### Pattern Recognition Accuracy of Replenished Items in Study 3

For the four-dimensional item pool, the pattern recognition performance in CSR for the LPRM-GR is listed in Table 9. It showed that the CSR values with estimated abilities were close to their benchmarks in most scenarios. Furthermore, when the weighted BIC was used to choose the optimal item-trait patterns, the accuracy of the LPRM-GR was significantly improved, which is particularly evident for the conditions with correlated abilities. In general, comparing the results from Tables 1, 5, 9, it can be observed that the weighted BIC assisted the LPRM-GR in producing higher recognition accuracy than the regular BIC, especially for the correlated-ability conditions.

**TABLE 9:** Correct specification rate of item-trait patterns identified by the LPRM-GR in Study 3.

Condition			LPRM-GR with ability estimates				LPRM-GR with true abilities	
				
			Regular BIC	Weighted BIC			Regular BIC (%)	Weighted BIC (%)
	
*M*+1	Latent trait correlation	Test length type	Item selection criterion					
			
			Bayesian A-optimality (%)	Bayesian D-optimality (%)	Bayesian A-optimality (%)	Bayesian D-optimality (%)		
3	Independent	Fixed-length	99.32	99.48	99.83	99.68	99.35	99.98
		Variable-length	99.32	99.38	99.95	99.92		
	Correlated	Fixed-length	87.65	87.05	93.93	93.68	98.57	98.70
		Variable-length	90.73	90.35	95.38	95.20		
4	Independent	Fixed-length	99.20	99.12	99.93	99.90	99.42	100
		Variable-length	99.28	99.30	99.97	99.97		
	Correlated	Fixed-length	86.87	87.28	93.13	93.13	98.38	98.70
		Variable-length	90.32	89.93	94.58	94.57		

Similar to the results in Tables 1, 5, Table 9 also showed that the CSRs for the correlated-ability conditions were lower than those for the independent-ability conditions. In addition, the CSR values for the variable-length tests were generally higher than those for the fixed-length tests, which was especially noticeable for the correlated-ability conditions. In contrast to the results with similar conditions in Tables 1, 5, the CSRs of the LPRM-GR were not significantly sensitive to the increase of dimensionality.

#### Ability Parameter Estimation Comparison in Multidimensional Computerized Adaptive Testing of Study 3

The recovery of ability parameters in Study 3 is listed in Table 10. It was found that the ability estimation accuracy for Bayesian A-Optimality was slightly better than that for Bayesian D-Optimality; the AME and RMSE values for the estimated correlated abilities were better than those for the independent abilities. Also, the variable-length tests in this study produced more precise estimates with respect to abilities than the fixed-length tests.

**TABLE 10:** Recovery of ability parameters in Study 3.

Condition			Item selection criterion			
	
			Bayesian A-optimality		Bayesian D-optimality	
				
*M*+1	Latent trait correlation	Test length type	AME (θ)	RMSE (θ)	AME (θ)	RMSE (θ)
3	Independent	Fixed-length	0.4650	0.5832	0.4706	0.5903
		Variable-length	0.3858	0.4839	0.3888	0.4876
	Correlated	Fixed-length	0.4189	0.5258	0.4220	0.5298
		Variable-length	0.3602	0.4519	0.3627	0.4551
4	Independent	Fixed-length	0.4528	0.5679	0.4576	0.5738
		Variable-length	0.3719	0.4664	0.3749	0.4020
	Correlated	Fixed-length	0.4099	0.5144	0.4133	0.5188
		Variable-length	0.3494	0.4383	0.3515	0.4409

#### Item Parameter Estimation Accuracy of Replenished Items in Study 3

Tables 11, 12 list the AME values of item parameters of replenished items in study 3, which were estimated based on the optimal item-trait patterns selected by the LPRM-GR. For the four-dimensional replenished items, it can be inferred from Tables 9, 11, 12 that the estimation accuracy of intercept parameters was less sensitive to the pattern recognition accuracy than the discrimination parameters. Furthermore, the recovery of estimated discrimination parameters based on the patterns identified by the LPRM-GR performs better with weighted BIC than that with the regular BIC, while the recovery of estimated intercept parameters performs similarly for the LPRM-GR with the two types of BIC.

**TABLE 11:** Recovery of item parameters based on patterns from the LPRM-GR with ability estimates and regular BIC in Study 3.

Condition			LPRM-GR with ability estimates			
	
*M*+1	Latent trait correlation	Test length type	Item selection criterion			
			
			Bayesian A-optimality		Bayesian D-optimality	
				
			AME (*a*)	AME (*b*)	AME (*a*)	AME (*b*)
3	Independent	Fixed-length	0.0630	0.1962	0.0634	0.1981
		Variable-length	0.0534	0.1604	0.0532	0.1615
	Correlated	Fixed-length	0.1401	0.1646	0.1428	0.1654
		Variable-length	0.1097	0.1438	0.1113	0.1442
4	Independent	Fixed-length	0.0594	0.1917	0.0602	0.1939
		Variable-length	0.0486	0.1542	0.0488	0.1550
	Correlated	Fixed-length	0.1373	0.1544	0.1371	0.1558
		Variable-length	0.1057	0.1338	0.1069	0.1339

**TABLE 12:** Recovery of item parameters based on patterns from the LPRM-GR with ability estimates and weighted BIC in Study 3.

Condition			LPRM-GR with ability estimates			
	
*M*+1	Latent trait correlation	Test length type	Item selection criterion			
			
			Bayesian A-optimality		Bayesian D-optimality	
				
			AME (*a*)	AME (*b*)	AME (*a*)	AME (*b*)
3	Independent	Fixed-length	0.0620	0.1970	0.0637	0.1989
		Variable-length	0.0522	0.1606	0.0523	0.1618
	Correlated	Fixed-length	0.1128	0.1679	0.1139	0.1690
		Variable-length	0.0908	0.1453	0.0916	0.1458
4	Independent	Fixed-length	0.0577	0.1921	0.0585	0.1944
		Variable-length	0.0472	0.1544	0.0475	0.1553
	Correlated	Fixed-length	0.1120	0.1570	0.1131	0.1582
		Variable-length	0.0890	0.1351	0.0889	0.1353

Moreover, the comparison of the AMEs among the three simulation studies illustrated that the discrimination parameter estimation accuracy was obviously affected by the pattern recognition accuracy of the LPRM-GR, especially for the conditions with correlated abilities. Results of the three studies also showed that the AMEs of item parameters based on the MCAT under the variable-length rule were lower than those for the fixed-length rule.

## Conclusion and Discussion

To manually identify how the replenished items in MCAT based on MGRM measure the latent traits is not only time-consuming but also affecting the accuracy and quality of the MCAT. In contrast, to automatically detect the item-trait patterns for replenished items with graded responses in the MCAT item pool can facilitate the item calibration of the items and benefit for the security of the subsequent diagnostic information mining and feedback. At the basis of Sun and Ye (2019), but not limited to, this research proposes a data-driven pattern recognition method for graded-response items, LPRM-GR, to find the optimal item-trait patterns of replenished items fitted by the MGRM in MCAT, which extends the framework of the LPRM to the case of graded responses for the item pool replenishment. The LPRM-GR formulates the L_1_-regularized optimization and gives the coordinate descent algorithm to solve it, and investigate the performance of the regular BIC and the weighted BIC, respectively, for selecting the optimal patterns of replenished items from the alternatives. Three simulation studies were conducted to evaluate the LPRM-GR with the two types of BIC in multiple cases across different numbers of response categories, ability correlation, stopping rules, and item selection criteria.

### Conclusion

Conclusions can be drawn from the results of three studies in simulation. In general, if one focuses on the CSR of item-trait patterns and the AME of estimated item parameters, it can be found: the LPRM-GR for variable-length scenarios performed better than that for fixed-length scenarios; the proposed method for independent cases performed better than that for correlated cases in terms of ability correlation; the proposed method with the Bayesian A-optimality in MCAT performed as similar as that with the Bayesian D-optimality; the proposed method for the MGRM with three response categories and that with four response categories had similar results; the higher pattern recognition accuracy of the LPRM-GR yielded comparatively higher estimation accuracy of discrimination parameters in MGRM; the weighted BIC performed better than the regular BIC for assisting the LPRM-GR in finding the optimal patterns. In addition, the estimation accuracy of discrimination parameters in MGRM was higher than that of intercept parameters.

Details of the findings of the three studies are given as follows. Firstly, the CSRs of the selected optimal patterns for the LPRM-GR with ability estimates in the MCAT procedure reached 86.87–100% for the overall cases for the three numbers of dimensionality. Specifically, the CSRs of the LPRM-GR with the ability estimates under the overall conditions are above 86%; for the two- and three-dimensional item pools, the CSRs are above 98% and 94%. By comparison with the benchmarks, the CSRs indicate that it is feasible for the LPRM-GR to help the LASSO optimization procedure by using ability estimates from the MCAT. Since it is not possible to know examinees’ true abilities in practice, with the appropriate sample size designed in simulation, the ability estimates ensure that the LPRM-GR can detect the optimal item-trait patterns of graded-response items in relatively satisfactory accuracy and efficiency.

Secondly, a worth-noting result is that the LPRM-GR with ability estimates and the weighted BIC obtains fairly high recognition accuracy, for which the CSRs reach over 93% for the overall cases and even being close to 100% for some cases. Regarding the overall performance of the LPRM-GR with the two types of BIC in pattern recognition accuracy and item parameter recovery, the studies demonstrate that the weighted BIC can help LPRM-GR improve the recognition accuracy better than the regular BIC, especially in the conditions for correlated abilities. The weighted BIC used in simulation has conveniently calculated weights, which provides a good choice for the practical application of the LPRM-GR.

Thirdly, taking an insight into the LPRM-GR with the weighted BIC, it can be found that: for the correlated ability conditions, the MCAT with SE rule and the constraint of up to 100 items in the LPRM-GR produces significantly better ability estimates, but slightly better CSRs, than the fixed-length rule (i.e., 50-length). The variable-length rule used by the LPRM-GR can play the positive part in ability estimation accuracy, which consequently to some extent benefits pattern recognition accuracy and discrimination parameter recovery of the replenished items with graded responses. As a matter of fact, it can be found that for most simulation cases with the variable-length rule, the length of MCAT in LPRM-GR is larger than 50, which could primarily explain the yield of better ability estimates. Nevertheless, the three studies also show that for the independent ability conditions, the fixed-length rule used in the LPRM-GR can provide similar pattern recognition accuracy to the variable-length rule, although the ability estimation accuracy for the former is not as good as the latter. In practice, if test developers cannot ignore the possible low computing efficiency yielded by the variable-length rule, the fixed-length MCAT can be a good choice for the application of the LPRM-GR.

Fourthly, for the two types of response categories of MGRM, the results in three studies suggest that the LPRM-GR can both get relatively high CSRs under the designed conditions, so by simulation, the response-category number has a relatively slight influence on ability estimation accuracy, pattern recognition accuracy, and item parameter recovery.

### Discussion

Advantages of the LPRM-GR are illustrated as follows. As one of the key parts for properly maintaining the functioning of the MCAT system, item-trait pattern recognition for item replenishment of the tests with polytomous responses is necessary. As the polytomous extension of the LPRM, the LPRM-GR can solve the recognition problem by detecting the optimal item-trait patterns of replenished items with graded responses efficiently and accurately, which appropriately utilizes the MCAT operation to integrate with the LASSO and BIC to promote the data-driven pattern recognition. Additionally, the LPRM-GR addresses the potential meaning of examinees’ item scoring frequencies in developing the weighted BIC for the further improvement of the effect of selecting the optimal patterns based on the LASSO. The advantage of the weighted BIC utilized in the LPRM-GR is well-supported by the results of the studies, especially for the simulation cases of correlated abilities.

As pointed out by Sun and Ye (2019), although the online calibration methods (e.g., Chen and Wang, 2016; Chen et al., 2017) and the LPRM can utilize the online feature of the MCAT, they solve different problems. An online calibration method focuses on estimating item parameters of replenished items based on well-known patterns, which can be reached by either a theory-driven or a data-driven method. The LPRM and LPRM-GR take interest in selecting the optimal pattern for each replenished item from all the possible alternatives, i.e., considering statistical goodness-of-fit for items based on the penalized-likelihood optimization like the L_1_-regularization. Similar to the advantages of the LPRM, the MCAT online function used in Step 1 of the LPRM-GR can automatically put the item parameters of the replenished items on the same scale as the operational ones in the item pool, which saves time and labor costs for the pretest of the replenished items. For a considerable long period of MCAT operation, the number of replenished items in the item pool can be relatively large, the data-driven methods may save the recognition efficiency to some extent. Moreover, the MCAT designed for the LPRM-GR does not require an extremely large sample size. Certainly, if the practical situation permits, an appropriate increase in the sample size for the proposed method may still expectedly yield higher pattern recognition accuracy. Too long tests for the variable-length rule can increase the fatigue effect as well as time costs, so for the LPRM-GR, which type of stopping rules should be put into practice needs full consideration of the tradeoff between pattern recognition accuracy and efficiency for estimating abilities.

Taking insight into the sample size of the LPRM-GR in simulation, it can be found that 4,000 examinees are designed for the graded-response scenario. The purpose of designing 800 examinees for each MCAT of the LPRM-GR is to fully consider the relatively more item parameters for the MGRM than that for the M2PLM, so that to investigate how well the LPRM-GR performs in pattern recognition accuracy in a relatively large sample size for the MGRM. Simulation results sufficiently indicate the above sample size can help the LPRM-GR getting good results in comparison with the benchmarks. Note that the sample size here is not too large to implement for the LPRM-GR, so based on the above considerations, the LPRM-GR is expected to be feasible and have good application value.

Implications and future directions are summarized as follows. First of all, this research takes observed responses as the role of the so-called adaptive weighting of the likelihood (e.g., Hu and Zidek, 2002) in BIC, and found that the weighted BIC exploited in this paper had even better performance than the regular BIC. Those weights for the weighted BIC are also easy to compute. Note that Warm (1989) propose a weighted likelihood estimator for ability estimation in the three-parameter logistic model. The idea for defining weights of the weighted likelihood is technically to measure the amount of information by the Fisher’s information function with abilities. Similar ideas were subsequently generalized to compensatory MIRT models (e.g., Tseng and Hsu, 2001; Wang, 2015). The idea of measuring the item information by the statistical variant of Fisher information matrix can also be found in constructing multidimensional item selection indices (e.g., Mulder and van der Linden, 2009). Thus, if thinking about other forms of the weights in the likelihood for constructing the adaptive weighted BIC, reasonable variants such as the determinant, trace, or other math quantities composed of examinees’ Fisher information may be worth experimenting and discussing in the future. Secondly, it is possible to take into account other shrinkage methods for selecting appropriate item-trait patterns of replenished items such as the elastic net (Zou and Hastie, 2005) and the adaptive LASSO (Zou, 2006) in conjunction with the proposed approach in future. Future research can also consider further pattern recognition study for more test situations or some extended models. For instance, the proposed method can also be extended for the items fitted by other MIRT models, such as the bi-factor models (Gibbons and Hedeker, 1992; Gibbons et al., 2007; Seo and Weiss, 2015), the two-tier full-information item factor model (Cai, 2010), and hierarchical test situations.

In addition, a limitation of the LPRM-GR is that its idea for pattern recognition constructs on the compensatory MIRT model with graded responses, so how to extend that to suit for the non-compensatory MIRT models can be considered in the future. The current pattern recognition method can also be extended to design for more variable-length stopping rules (e.g., Wang et al., 2013; Wang et al., 2018b) or new item selection and exposure control methods (e.g., Chen et al., 2020) in MCAT. As reviewers’ suggestion, researchers can furtherly explore the practical topics on the MCAT item pool replenishment in future: what indices should be comprehensively considered for finding the frequently used operational items; how long items should be used before item exposure becomes a concern; what specific factors that test developers should be aware for the MCAT development.

## Data Availability Statement

The raw data supporting the conclusions of this article will be made available by the corresponding author upon request, without undue reservation.

## Author Contributions

JS was in charge of the literature review, original idea and derivation of methodology, simulation design and computing codes, and drafting and revising of the manuscript. ZY contributed to the literature review, application of the weighted BIC, computing codes, program running, tabular data collection, and drafting of the manuscript. LR contributed to the literature review, computing codes, and drafting and revising of the manuscript. JL contributed to the literature review and revising of the manuscript. All authors contributed to the article and approved the submitted version.

## Conflict of Interest

The authors declare that the research was conducted in the absence of any commercial or financial relationships that could be construed as a potential conflict of interest.

## Publisher’s Note

All claims expressed in this article are solely those of the authors and do not necessarily represent those of their affiliated organizations, or those of the publisher, the editors and the reviewers. Any product that may be evaluated in this article, or claim that may be made by its manufacturer, is not guaranteed or endorsed by the publisher.

## Appendix

Derivation of solving the L_1_-regularized optimization in Equation (5) *via* the coordinate descent algorithm and Fisher’s scoring iteration is given as follows. For simplicity, new symbols of the *i*th examinee’s responses to the *j*th item (i.e., *Y*_*ij*_) in Equation (2) are here redefined as (*y*_*ij*0_,…,*y*_*ijM*_), where *y*_*ijm*_ ∈ {0,1} and $\sum_{m=0}^{M}y_{i\,j\,m}=1$. If Y_*ij*_ = *m*, then *y*_*ijm*_ = 1 and $y_{i\,j\,\tilde{m}}=0\left(\tilde{m}\neq m\right)$. For simplicity, the symbol θ_*i*_ is omitted from *P*_*ijm*_(θ_*i*_) and $P_{i\,j\,m}^{*}\,\left(\theta_{i}\right)$, which are hereafter denoted as *P*_*ijm*_ and $P_{i\,j\,m}^{*}$. The negative log-likelihood of the observed data and the known abilities based on the MGRM is

(15)
$$-l\,\left({\text{b}}_{j},{\text{a}}_{j},{\text{Y}}_{j},\Theta \right)=-\sum_{i=1}^{N}\log\left(\prod_{m=0}^{M}P_{i\,j\,m}^{y_{i\,j\,m}}\right)=-\sum_{i=1}^{N}\sum_{m=0}^{M}y_{i\,j\,m}\,\log\left(P_{i\,j\,m}\right).$$

Note that for the MGRM, the intercept parameters should be updated are *b*_*j*1_,…,*b*_*j*(*M*−1)_. By the definition of $\Delta \,P_{i\,j\,m}^{*}=P_{i\,j\,m}^{*}\,\left(1-P_{i\,j\,m}^{*}\right)$ and $\Delta^{2}\,P_{i\,j\,m}^{*}=P_{i\,j\,m}^{*}\,\left(1-P_{i\,j\,m}^{*}\right)\,\left(1-2\,P_{i\,j\,m}^{*}\right)$, the first-order derivative of Equation (15) with respect to *b*_*jm*_(*m* = 1,…,*M*−1) is represented as $g_{j\,m}=-\frac{\partial l\,\left(b_{j},a_{j},Y_{j},\Theta \right)}{\partial b_{j\,m}}$; after the *t*th iteration for item parameters, we have

(16)
$$g_{j\,m}^{\left(t\right)}=\sum_{i=1}^{N}\left[\frac{y_{i\,j\,\left(m-1\right)}}{P_{i\,j\,\left(m-1\right)}^{\left(t\right)}}\,\Delta \,P_{i\,j\,m}^{*\left(t\right)}-\frac{y_{i\,j\,m}}{P_{i\,j\,m}^{\left(t\right)}}\,\Delta \,P_{i\,j\,m}^{*\left(t\right)}\right].$$

For the Fisher’s scoring update, the second-order derivative of Equation (15) with respect to *b*_*jm*_ (i.e., $h_{j\,m}=-\frac{\partial^{2}l\,\left(b_{j},a_{j},Y_{j},\Theta \right)}{\partial b_{j\,m}^{2}}$) is substituted with its expectation, and then

(17)
$$E\,\left(h_{j\,m}^{\left(t\right)}\right)=-\sum_{i=1}^{N}\left[\left(\frac{1}{P_{i\,j\,\left(m-1\right)}^{\left(t\right)}}\,{\left(\Delta \,P_{i\,j\,m}^{*\left(t\right)}\right)}^{2}+\Delta^{2}\,P_{i\,j\,m}^{*\left(t\right)}\right)+\left(\frac{1}{P_{i\,j\,m}^{\left(t\right)}}\,{\left(\Delta \,P_{i\,j\,m}^{*\left(t\right)}\right)}^{2}-\Delta^{2}\,P_{i\,j\,m}^{*\left(t\right)}\right)\right].$$

The Fisher’s scoring update of *b*_*jm*_ for the (*t*+1)th iteration has the form:

(18)
$${\hat{b}}_{j\,m}^{\left(t+1\right)}={\hat{b}}_{j\,m}^{\left(t\right)}-\frac{g_{j\,m}^{\left(t\right)}}{E\,\left(h_{j\,m}^{\left(t\right)}\right)}.$$

The first-order derivative of Equation (15) with respect to *a*_*jk*_(*k* = 1,…,*K*) is represented as $g_{j\,k}=-\frac{\partial l\,\left(b_{j},a_{j},Y_{j},\Theta \right)}{\partial a_{j\,k}}$, and then

(19)
$$g_{j\,k}^{\left(t\right)}=-\sum_{i=1}^{N}\sum_{m=0}^{M}\frac{y_{i\,j\,m}}{P_{i\,j\,m}^{\left(t\right)}}\,\left[\Delta \,P_{i\,j\,m}^{*\left(t\right)}-\Delta \,P_{i\,j\,\left(m+1\right)}^{*\left(t\right)}\right]\,\theta_{i\,k}.$$

The expected second-order derivative of Equation (15) of *a*_*jk*_ is expressed as

(20)
$$E\left(h_{j\,k}^{\left(t\right)}\right)=\sum_{i=1}^{N}\sum_{m=0}^{M}{\left\{\frac{1}{P_{i\,j\,m}^{\left(t\right)}}\text{[}\Delta P_{i\,j\,m}^{*\left(t\right)}-\Delta P_{i\,j\,\left(m+1\right)}^{*\left(t\right)}\right]}^{2}-\text{[}\Delta^{2}P_{i\,j\,m}^{*\left(t\right)}-\Delta^{2}P_{i\,j\,\left(m+1\right)}^{*\left(t\right)}]\}\theta {}_{i\,k}{}^{2}.$$

The coordinate-wise update of *a*_*jk*_ for the (*t*+1)th iteration has the form:

(21)
$${\hat{a}}_{j\,k}^{\left(t+1\right)}=S\,\left({\hat{a}}_{j\,k}^{\left(t\right)}-\frac{g_{j\,k}^{\left(t\right)}}{E\,\left(h_{j\,k}^{\left(t\right)}\right)},\lambda \right),$$

where the soft-thresholding operator (e.g., Friedman et al., 2010) is

(22)
$$\text{S}\,\left(z,\gamma \right)=\left\{ \begin{array}{cc} z-\gamma \; & i\,f\,z>0\,a\,n\,d\,\gamma <\left|z\right|\\ z+\gamma \; & i\,f\,z<0\,a\,n\,d\,\gamma <\left|z\right|\\ 0\; & i\,f\,\gamma \geq \left|z\right| \end{array}\right..$$
